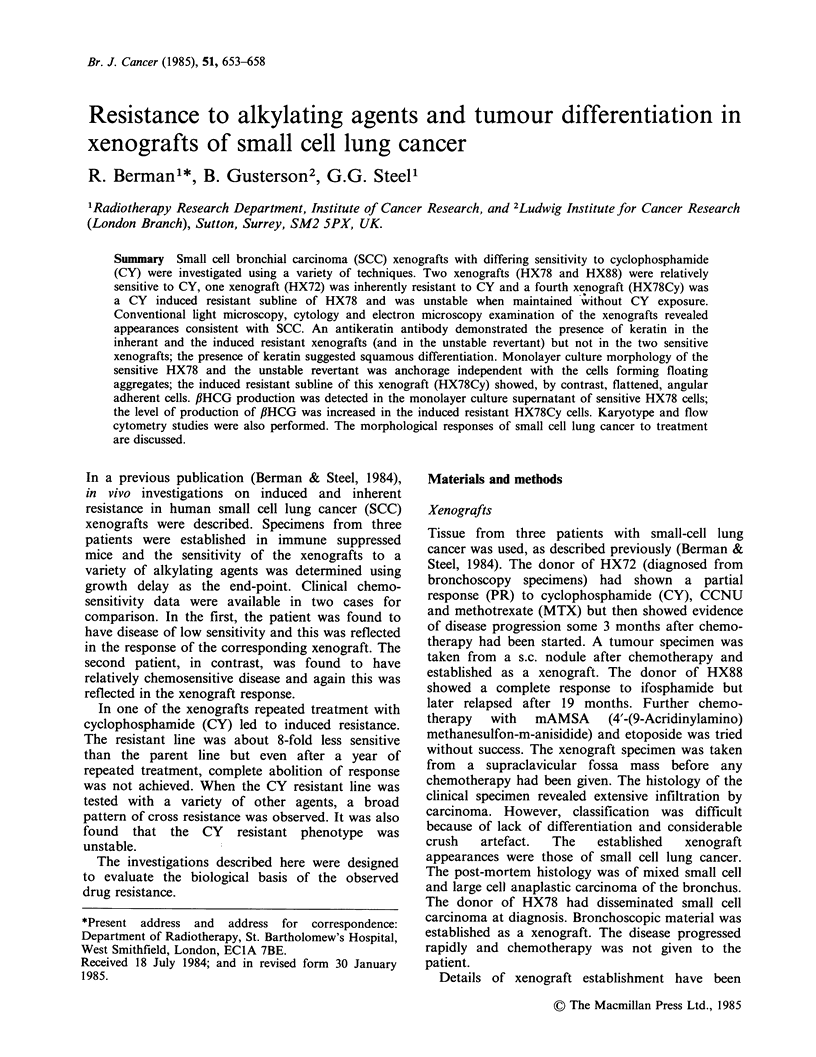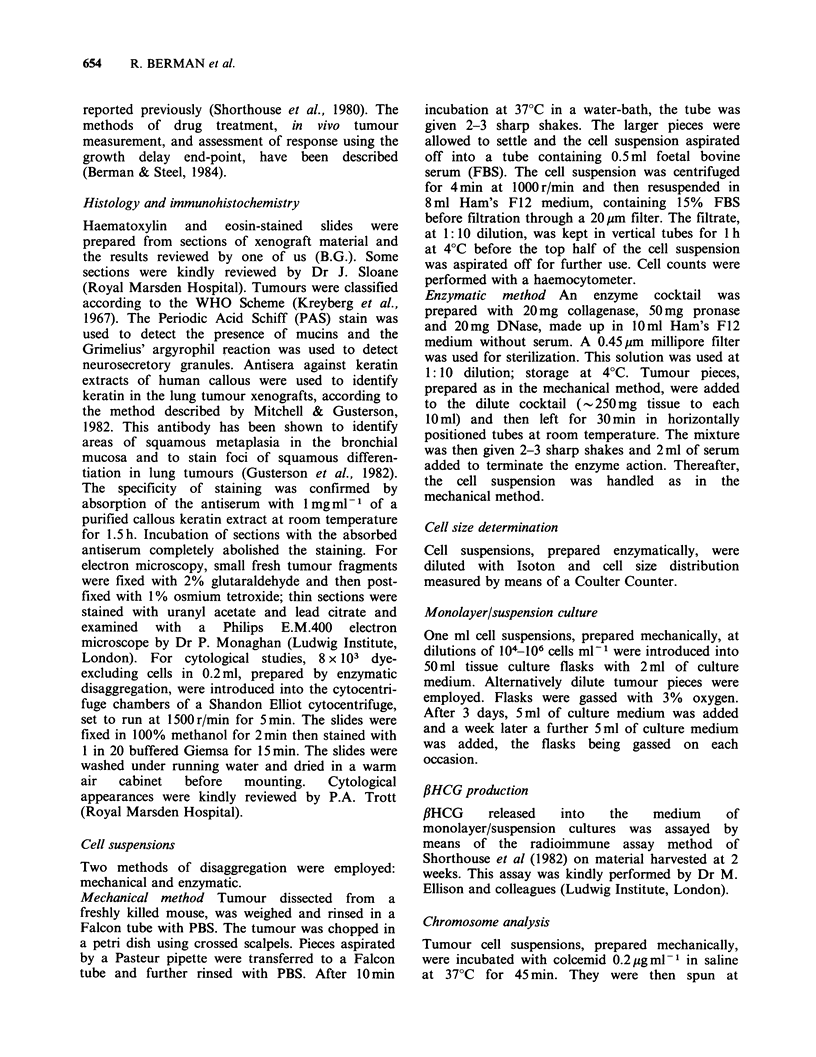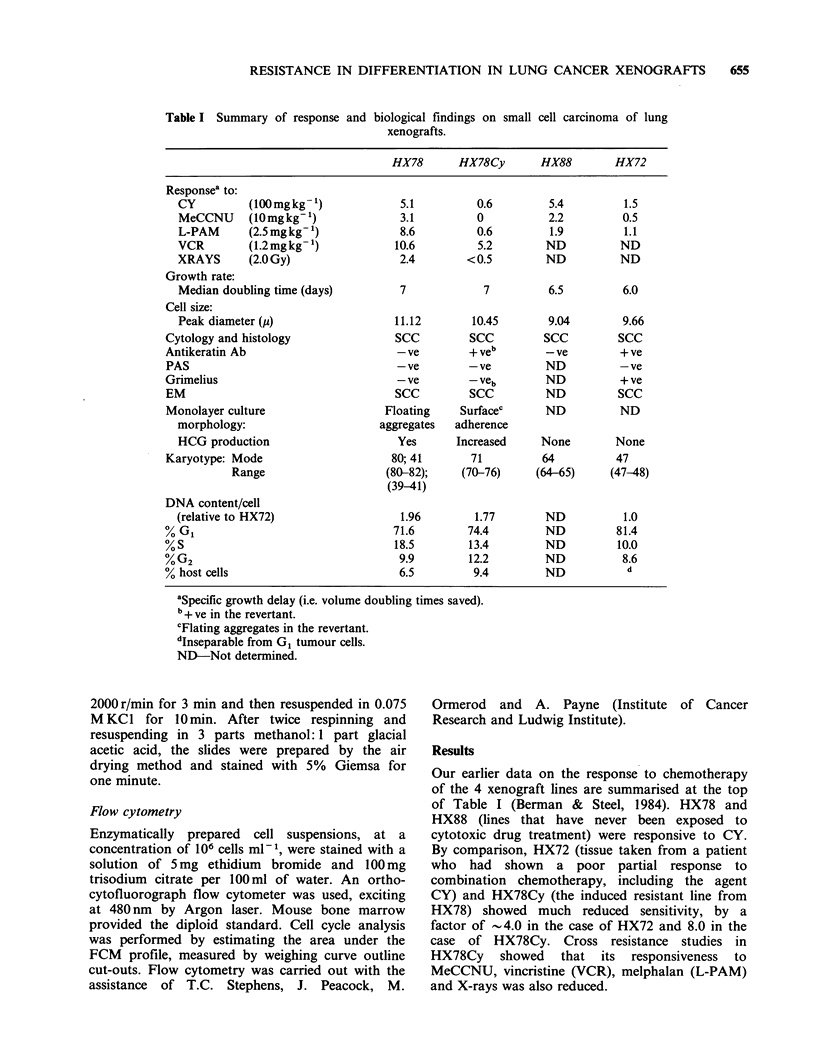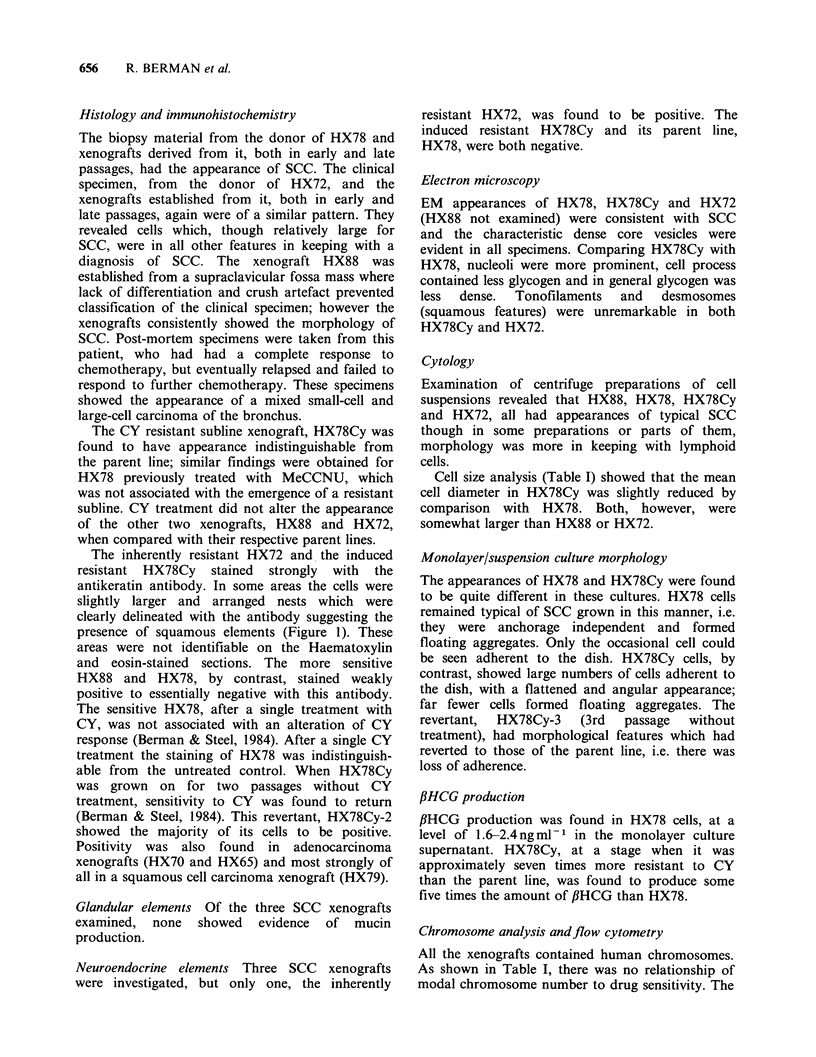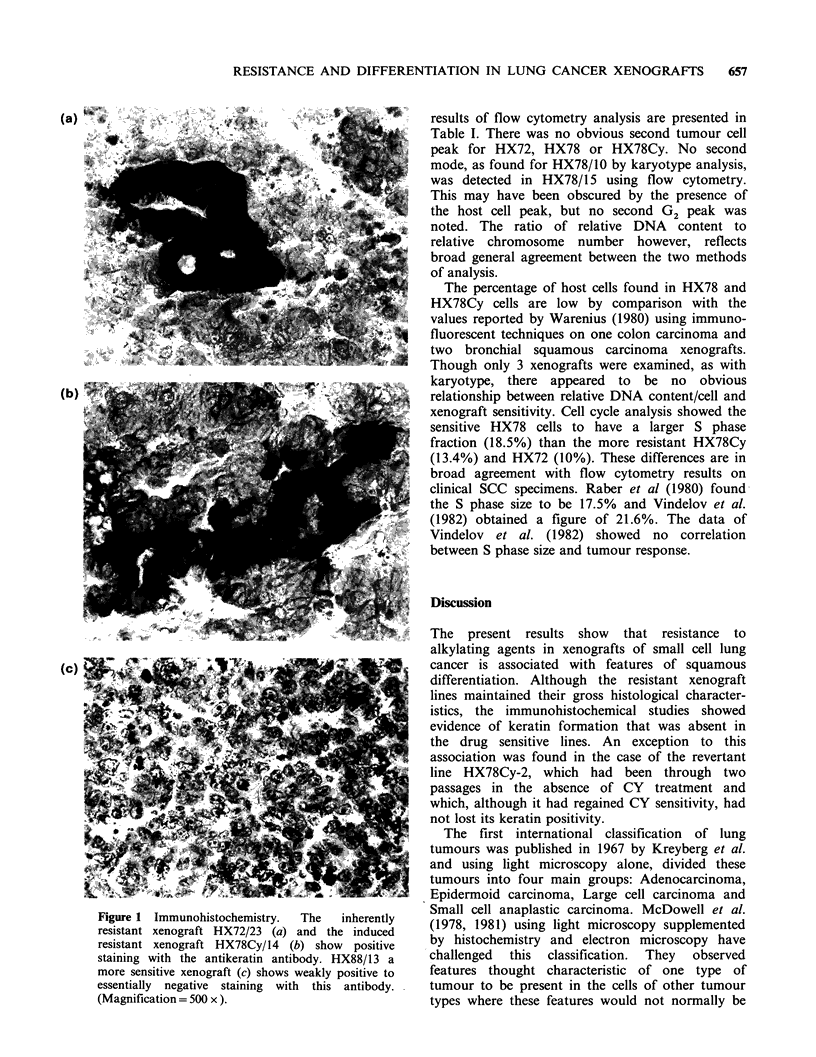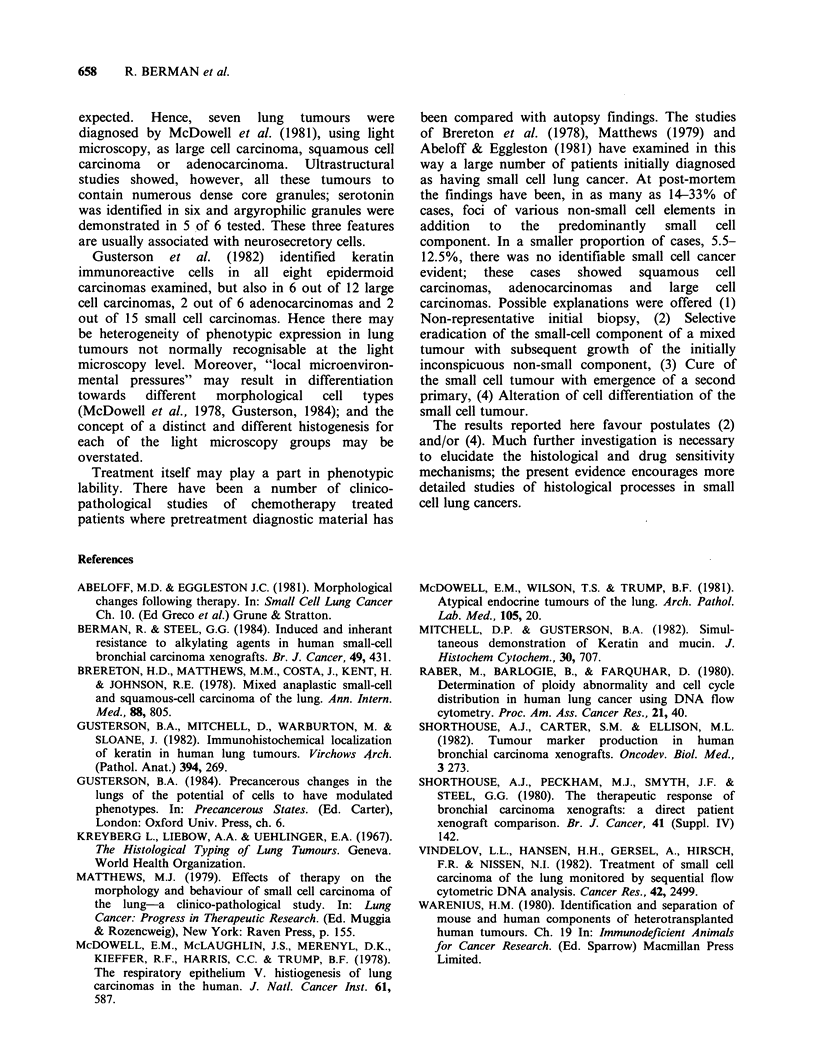# Resistance to alkylating agents and tumour differentiation in xenografts of small cell lung cancer.

**DOI:** 10.1038/bjc.1985.98

**Published:** 1985-05

**Authors:** R. Berman, B. Gusterson, G. G. Steel

## Abstract

**Images:**


*Br. J. Cancer* (1985), **51**, 653–658

### Resistance to alkylating agents and tumour differentiation in xenografts of small cell lung cancer

R. Berman[1]*, B. Gusterson[2], G.G. Steel[1]

[1]*Radiotherapy Research Department, Institute of Cancer Research, and* [2]*Ludwig Institute for Cancer Research (London Branch), Sutton, Surrey, SM2 5PX, UK.*

**Summary** Small cell bronchial carcinoma (SCC) xenografts with differing sensitivity to cyclophosphamide (CY) were investigated using a variety of techniques. Two xenografts (HX78 and HX88) were relatively sensitive to CY, one xenograft (HX72) was inherently resistant to CY and a fourth xenograft (HX78Cy) was a CY induced resistant subline of HX78 and was unstable when maintained without CY exposure. Conventional light microscopy, cytology and electron microscopy examination of the xenografts revealed appearances consistent with SCC. An antikeratin antibody demonstrated the presence of keratin in the inherant and the induced resistant xenografts (and in the unstable revertant) but not in the two sensitive xenografts; the presence of keratin suggested squamous differentiation. Monolayer culture morphology of the sensitive HX78 and the unstable revertant was anchorage independent with the cells forming floating aggregates; the induced resistant subline of this xenograft (HX78Cy) showed, by contrast, flattened, angular adherent cells. βHCG production was detected in the monolayer culture supernatant of sensitive HX78 cells; the level of production of βHCG was increased in the induced resistant HX78Cy cells. Karyotype and flow cytometry studies were also performed. The morphological responses of small cell lung cancer to treatment are discussed.

In a previous publication (Berman & Steel, 1984), *in vivo* investigations on induced and inherent resistance in human small cell lung cancer (SCC) xenografts were described. Specimens from three patients were established in immune suppressed mice and the sensitivity of the xenografts to a variety of alkylating agents was determined using growth delay as the end-point. Clinical chemosensitivity data were available in two cases for comparison. In the first, the patient was found to have disease of low sensitivity and this was reflected in the response of the corresponding xenograft. The second patient, in contrast, was found to have relatively chemosensitive disease and again this was reflected in the xenograft response.

In one of the xenografts repeated treatment with cyclophosphamide (CY) led to induced resistance. The resistant line was about 8-fold less sensitive than the parent line but even after a year of repeated treatment, complete abolition of response was not achieved. When the CY resistant line was tested with a variety of other agents, a broad pattern of cross resistance was observed. It was also found that the CY resistant phenotype was unstable.

The investigations described here were designed to evaluate the biological basis of the observed drug resistance.

*Present address and address for correspondence: Department of Radiotherapy, St. Bartholomew's Hospital, West Smithfield, London, EC1A 7BE.

Received 18 July 1984; and in revised form 30 January 1985.

### Materials and methods

### *Xenografts*

Tissue from three patients with small-cell lung cancer was used, as described previously (Berman & Steel, 1984). The donor of HX72 (diagnosed from bronchoscopy specimens) had shown a partial response (PR) to cyclophosphamide (CY), CCNU and methotrexate (MTX) but then showed evidence of disease progression some 3 months after chemotherapy had been started. A tumour specimen was taken from a s.c. nodule after chemotherapy and established as a xenograft. The donor of HX88 showed a complete response to ifosphamide but later relapsed after 19 months. Further chemotherapy with mAMSA (4′-(9-Acridinylamino) methanesulfon-m-anisidide) and etoposide was tried without success. The xenograft specimen was taken from a supraclavicular fossa mass before any chemotherapy had been given. The histology of the clinical specimen revealed extensive infiltration by carcinoma. However, classification was difficult because of lack of differentiation and considerable crush artefact. The established xenograft appearances were those of small cell lung cancer. The post-mortem histology was of mixed small cell and large cell anaplastic carcinoma of the bronchus. The donor of HX78 had disseminated small cell carcinoma at diagnosis. Bronchoscopic material was established as a xenograft. The disease progressed rapidly and chemotherapy was not given to the patient.

Details of xenograft establishment have been

© The Macmillan Press Ltd., 1985

reported previously (Shorthouse *et al.*, 1980). The methods of drug treatment, *in vivo* tumour measurement, and assessment of response using the growth delay end-point, have been described (Berman & Steel, 1984).

*Histology and immunohistochemistry*

Haematoxylin and eosin-stained slides were prepared from sections of xenograft material and the results reviewed by one of us (B.G.). Some sections were kindly reviewed by Dr J. Sloane (Royal Marsden Hospital). Tumours were classified according to the WHO Scheme (Kreyberg *et al.*, 1967). The Periodic Acid Schiff (PAS) stain was used to detect the presence of mucins and the Grimelius' argyrophil reaction was used to detect neurosecretory granules. Antisera against keratin extracts of human callous were used to identify keratin in the lung tumour xenografts, according to the method described by Mitchell & Gusterson, 1982. This antibody has been shown to identify areas of squamous metaplasia in the bronchial mucosa and to stain foci of squamous differentiation in lung tumours (Gusterson *et al.*, 1982). The specificity of staining was confirmed by absorption of the antiserum with $1\,mg\,ml^{-1}$ of a purified callous keratin extract at room temperature for 1.5 h. Incubation of sections with the absorbed antiserum completely abolished the staining. For electron microscopy, small fresh tumour fragments were fixed with 2% glutaraldehyde and then post-fixed with 1% osmium tetroxide; thin sections were stained with uranyl acetate and lead citrate and examined with a Philips E.M.400 electron microscope by Dr P. Monaghan (Ludwig Institute, London). For cytological studies, $8 \times 10^3$ dye-excluding cells in 0.2 ml, prepared by enzymatic disaggregation, were introduced into the cytocentrifuge chambers of a Shandon Elliot cytocentrifuge, set to run at 1500 r/min for 5 min. The slides were fixed in 100% methanol for 2 min then stained with 1 in 20 buffered Giemsa for 15 min. The slides were washed under running water and dried in a warm air cabinet before mounting. Cytological appearances were kindly reviewed by P.A. Trott (Royal Marsden Hospital).

*Cell suspensions*

Two methods of disaggregation were employed: mechanical and enzymatic.

*Mechanical method* Tumour dissected from a freshly killed mouse, was weighed and rinsed in a Falcon tube with PBS. The tumour was chopped in a petri dish using crossed scalpels. Pieces aspirated by a Pasteur pipette were transferred to a Falcon tube and further rinsed with PBS. After 10 min incubation at 37°C in a water-bath, the tube was given 2–3 sharp shakes. The larger pieces were allowed to settle and the cell suspension aspirated off into a tube containing 0.5 ml foetal bovine serum (FBS). The cell suspension was centrifuged for 4 min at 1000 r/min and then resuspended in 8 ml Ham's F12 medium, containing 15% FBS before filtration through a 20 μm filter. The filtrate, at 1:10 dilution, was kept in vertical tubes for 1 h at 4°C before the top half of the cell suspension was aspirated off for further use. Cell counts were performed with a haemocytometer.

*Enzymatic method* An enzyme cocktail was prepared with 20 mg collagenase, 50 mg pronase and 20 mg DNase, made up in 10 ml Ham's F12 medium without serum. A 0.45 μm millipore filter was used for sterilization. This solution was used at 1:10 dilution; storage at 4°C. Tumour pieces, prepared as in the mechanical method, were added to the dilute cocktail (~250 mg tissue to each 10 ml) and then left for 30 min in horizontally positioned tubes at room temperature. The mixture was then given 2–3 sharp shakes and 2 ml of serum added to terminate the enzyme action. Thereafter, the cell suspension was handled as in the mechanical method.

*Cell size determination*

Cell suspensions, prepared enzymatically, were diluted with Isoton and cell size distribution measured by means of a Coulter Counter.

*Monolayer/suspension culture*

One ml cell suspensions, prepared mechanically, at dilutions of $10^4$–$10^6$ cells $ml^{-1}$ were introduced into 50 ml tissue culture flasks with 2 ml of culture medium. Alternatively dilute tumour pieces were employed. Flasks were gassed with 3% oxygen. After 3 days, 5 ml of culture medium was added and a week later a further 5 ml of culture medium was added, the flasks being gassed on each occasion.

*βHCG production*

βHCG released into the medium of monolayer/suspension cultures was assayed by means of the radioimmune assay method of Shorthouse *et al* (1982) on material harvested at 2 weeks. This assay was kindly performed by Dr M. Ellison and colleagues (Ludwig Institute, London).

*Chromosome analysis*

Tumour cell suspensions, prepared mechanically, were incubated with colcemid $0.2\,\mu g\,ml^{-1}$ in saline at 37°C for 45 min. They were then spun at

**Table I** Summary of response and biological findings on small cell carcinoma of lung xenografts.

| | *HX78* | *HX78Cy* | *HX88* | *HX72* |
|---|---|---|---|---|
| Response[a] to: | | | | |
| CY (100 mg kg$^{-1}$) | 5.1 | 0.6 | 5.4 | 1.5 |
| MeCCNU (10 mg kg$^{-1}$) | 3.1 | 0 | 2.2 | 0.5 |
| L-PAM (2.5 mg kg$^{-1}$) | 8.6 | 0.6 | 1.9 | 1.1 |
| VCR (1.2 mg kg$^{-1}$) | 10.6 | 5.2 | ND | ND |
| XRAYS (2.0 Gy) | 2.4 | <0.5 | ND | ND |
| Growth rate: | | | | |
| Median doubling time (days) | 7 | 7 | 6.5 | 6.0 |
| Cell size: | | | | |
| Peak diameter ($\mu$) | 11.12 | 10.45 | 9.04 | 9.66 |
| Cytology and histology | SCC | SCC | SCC | SCC |
| Antikeratin Ab | −ve | +ve[b] | −ve | +ve |
| PAS | −ve | −ve | ND | −ve |
| Grimelius | −ve | −ve[b] | ND | +ve |
| EM | SCC | SCC | ND | SCC |
| Monolayer culture morphology: | Floating aggregates | Surface[c] adherence | ND | ND |
| HCG production | Yes | Increased | None | None |
| Karyotype: Mode | 80; 41 | 71 | 64 | 47 |
| Range | (80–82); (39–41) | (70–76) | (64–65) | (47–48) |
| DNA content/cell (relative to HX72) | 1.96 | 1.77 | ND | 1.0 |
| % $G_1$ | 71.6 | 74.4 | ND | 81.4 |
| % S | 18.5 | 13.4 | ND | 10.0 |
| % $G_2$ | 9.9 | 12.2 | ND | 8.6 |
| % host cells | 6.5 | 9.4 | ND | [d] |

[a]Specific growth delay (i.e. volume doubling times saved).
[b]+ve in the revertant.
[c]Flating aggregates in the revertant.
[d]Inseparable from $G_1$ tumour cells.
ND—Not determined.

2000 r/min for 3 min and then resuspended in 0.075 M KCl for 10 min. After twice respinning and resuspending in 3 parts methanol: 1 part glacial acetic acid, the slides were prepared by the air drying method and stained with 5% Giemsa for one minute.

*Flow cytometry*

Enzymatically prepared cell suspensions, at a concentration of $10^6$ cells ml$^{-1}$, were stained with a solution of 5 mg ethidium bromide and 100 mg trisodium citrate per 100 ml of water. An ortho-cytofluorograph flow cytometer was used, exciting at 480 nm by Argon laser. Mouse bone marrow provided the diploid standard. Cell cycle analysis was performed by estimating the area under the FCM profile, measured by weighing curve outline cut-outs. Flow cytometry was carried out with the assistance of T.C. Stephens, J. Peacock, M. Ormerod and A. Payne (Institute of Cancer Research and Ludwig Institute).

### Results

Our earlier data on the response to chemotherapy of the 4 xenograft lines are summarised at the top of Table I (Berman & Steel, 1984). HX78 and HX88 (lines that have never been exposed to cytotoxic drug treatment) were responsive to CY. By comparison, HX72 (tissue taken from a patient who had shown a poor partial response to combination chemotherapy, including the agent CY) and HX78Cy (the induced resistant line from HX78) showed much reduced sensitivity, by a factor of ~4.0 in the case of HX72 and 8.0 in the case of HX78Cy. Cross resistance studies in HX78Cy showed that its responsiveness to MeCCNU, vincristine (VCR), melphalan (L-PAM) and X-rays was also reduced.

*Histology and immunohistochemistry*

The biopsy material from the donor of HX78 and xenografts derived from it, both in early and late passages, had the appearance of SCC. The clinical specimen, from the donor of HX72, and the xenografts established from it, both in early and late passages, again were of a similar pattern. They revealed cells which, though relatively large for SCC, were in all other features in keeping with a diagnosis of SCC. The xenograft HX88 was established from a supraclavicular fossa mass where lack of differentiation and crush artefact prevented classification of the clinical specimen; however the xenografts consistently showed the morphology of SCC. Post-mortem specimens were taken from this patient, who had had a complete response to chemotherapy, but eventually relapsed and failed to respond to further chemotherapy. These specimens showed the appearance of a mixed small-cell and large-cell carcinoma of the bronchus.

The CY resistant subline xenograft, HX78Cy was found to have appearance indistinguishable from the parent line; similar findings were obtained for HX78 previously treated with MeCCNU, which was not associated with the emergence of a resistant subline. CY treatment did not alter the appearance of the other two xenografts, HX88 and HX72, when compared with their respective parent lines.

The inherently resistant HX72 and the induced resistant HX78Cy stained strongly with the antikeratin antibody. In some areas the cells were slightly larger and arranged nests which were clearly delineated with the antibody suggesting the presence of squamous elements (Figure 1). These areas were not identifiable on the Haematoxylin and eosin-stained sections. The more sensitive HX88 and HX78, by contrast, stained weakly positive to essentially negative with this antibody. The sensitive HX78, after a single treatment with CY, was not associated with an alteration of CY response (Berman & Steel, 1984). After a single CY treatment the staining of HX78 was indistinguishable from the untreated control. When HX78Cy was grown on for two passages without CY treatment, sensitivity to CY was found to return (Berman & Steel, 1984). This revertant, HX78Cy-2 showed the majority of its cells to be positive. Positivity was also found in adenocarcinoma xenografts (HX70 and HX65) and most strongly of all in a squamous cell carcinoma xenograft (HX79).

*Glandular elements* Of the three SCC xenografts examined, none showed evidence of mucin production.

*Neuroendocrine elements* Three SCC xenografts were investigated, but only one, the inherently resistant HX72, was found to be positive. The induced resistant HX78Cy and its parent line, HX78, were both negative.

*Electron microscopy*

EM appearances of HX78, HX78Cy and HX72 (HX88 not examined) were consistent with SCC and the characteristic dense core vesicles were evident in all specimens. Comparing HX78Cy with HX78, nucleoli were more prominent, cell process contained less glycogen and in general glycogen was less dense. Tonofilaments and desmosomes (squamous features) were unremarkable in both HX78Cy and HX72.

*Cytology*

Examination of centrifuge preparations of cell suspensions revealed that HX88, HX78, HX78Cy and HX72, all had appearances of typical SCC though in some preparations or parts of them, morphology was more in keeping with lymphoid cells.

Cell size analysis (Table I) showed that the mean cell diameter in HX78Cy was slightly reduced by comparison with HX78. Both, however, were somewhat larger than HX88 or HX72.

*Monolayer/suspension culture morphology*

The appearances of HX78 and HX78Cy were found to be quite different in these cultures. HX78 cells remained typical of SCC grown in this manner, i.e. they were anchorage independent and formed floating aggregates. Only the occasional cell could be seen adherent to the dish. HX78Cy cells, by contrast, showed large numbers of cells adherent to the dish, with a flattened and angular appearance; far fewer cells formed floating aggregates. The revertant, HX78Cy-3 (3rd passage without treatment), had morphological features which had reverted to those of the parent line, i.e. there was loss of adherence.

*βHCG production*

βHCG production was found in HX78 cells, at a level of 1.6–2.4 $\mathrm{ng\,ml^{-1}}$ in the monolayer culture supernatant. HX78Cy, at a stage when it was approximately seven times more resistant to CY than the parent line, was found to produce some five times the amount of βHCG than HX78.

*Chromosome analysis and flow cytometry*

All the xenografts contained human chromosomes. As shown in Table I, there was no relationship of modal chromosome number to drug sensitivity. The

**Figure 1** Immunohistochemistry. The inherently resistant xenograft HX72/23 (*a*) and the induced resistant xenograft HX78Cy/14 (*b*) show positive staining with the antikeratin antibody. HX88/13 a more sensitive xenograft (*c*) shows weakly positive to essentially negative staining with this antibody. (Magnification = 500 ×).

results of flow cytometry analysis are presented in Table I. There was no obvious second tumour cell peak for HX72, HX78 or HX78Cy. No second mode, as found for HX78/10 by karyotype analysis, was detected in HX78/15 using flow cytometry. This may have been obscured by the presence of the host cell peak, but no second $G_2$ peak was noted. The ratio of relative DNA content to relative chromosome number however, reflects broad general agreement between the two methods of analysis.

The percentage of host cells found in HX78 and HX78Cy cells are low by comparison with the values reported by Warenius (1980) using immunofluorescent techniques on one colon carcinoma and two bronchial squamous carcinoma xenografts. Though only 3 xenografts were examined, as with karyotype, there appeared to be no obvious relationship between relative DNA content/cell and xenograft sensitivity. Cell cycle analysis showed the sensitive HX78 cells to have a larger S phase fraction (18.5%) than the more resistant HX78Cy (13.4%) and HX72 (10%). These differences are in broad agreement with flow cytometry results on clinical SCC specimens. Raber *et al* (1980) found the S phase size to be 17.5% and Vindelov *et al.* (1982) obtained a figure of 21.6%. The data of Vindelov *et al.* (1982) showed no correlation between S phase size and tumour response.

### Discussion

The present results show that resistance to alkylating agents in xenografts of small cell lung cancer is associated with features of squamous differentiation. Although the resistant xenograft lines maintained their gross histological characteristics, the immunohistochemical studies showed evidence of keratin formation that was absent in the drug sensitive lines. An exception to this association was found in the case of the revertant line HX78Cy-2, which had been through two passages in the absence of CY treatment and which, although it had regained CY sensitivity, had not lost its keratin positivity.

The first international classification of lung tumours was published in 1967 by Kreyberg *et al.* and using light microscopy alone, divided these tumours into four main groups: Adenocarcinoma, Epidermoid carcinoma, Large cell carcinoma and Small cell anaplastic carcinoma. McDowell *et al.* (1978, 1981) using light microscopy supplemented by histochemistry and electron microscopy have challenged this classification. They observed features thought characteristic of one type of tumour to be present in the cells of other tumour types where these features would not normally be expected. Hence, seven lung tumours were diagnosed by McDowell *et al.* (1981), using light microscopy, as large cell carcinoma, squamous cell carcinoma or adenocarcinoma. Ultrastructural studies showed, however, all these tumours to contain numerous dense core granules; serotonin was identified in six and argyrophilic granules were demonstrated in 5 of 6 tested. These three features are usually associated with neurosecretory cells.

Gusterson *et al.* (1982) identified keratin immunoreactive cells in all eight epidermoid carcinomas examined, but also in 6 out of 12 large cell carcinomas, 2 out of 6 adenocarcinomas and 2 out of 15 small cell carcinomas. Hence there may be heterogeneity of phenotypic expression in lung tumours not normally recognisable at the light microscopy level. Moreover, "local microenvironmental pressures" may result in differentiation towards different morphological cell types (McDowell *et al.*, 1978, Gusterson, 1984); and the concept of a distinct and different histogenesis for each of the light microscopy groups may be overstated.

Treatment itself may play a part in phenotypic lability. There have been a number of clinico-pathological studies of chemotherapy treated patients where pretreatment diagnostic material has been compared with autopsy findings. The studies of Brereton *et al.* (1978), Matthews (1979) and Abeloff & Eggleston (1981) have examined in this way a large number of patients initially diagnosed as having small cell lung cancer. At post-mortem the findings have been, in as many as 14–33% of cases, foci of various non-small cell elements in addition to the predominantly small cell component. In a smaller proportion of cases, 5.5–12.5%, there was no identifiable small cell cancer evident; these cases showed squamous cell carcinomas, adenocarcinomas and large cell carcinomas. Possible explanations were offered (1) Non-representative initial biopsy, (2) Selective eradication of the small-cell component of a mixed tumour with subsequent growth of the initially inconspicuous non-small component, (3) Cure of the small cell tumour with emergence of a second primary, (4) Alteration of cell differentiation of the small cell tumour.

The results reported here favour postulates (2) and/or (4). Much further investigation is necessary to elucidate the histological and drug sensitivity mechanisms; the present evidence encourages more detailed studies of histological processes in small cell lung cancers.

### References

ABELOFF, M.D. & EGGLESTON J.C. (1981). Morphological changes following therapy. In: *Small Cell Lung Cancer* Ch. 10. (Ed Greco *et al.*) Grune & Stratton.

BERMAN, R. & STEEL, G.G. (1984). Induced and inherant resistance to alkylating agents in human small-cell bronchial carcinoma xenografts. *Br. J. Cancer*, **49**, 431.

BRERETON, H.D., MATTHEWS, M.M., COSTA, J., KENT, H. & JOHNSON, R.E. (1978). Mixed anaplastic small-cell and squamous-cell carcinoma of the lung. *Ann. Intern. Med.*, **88**, 805.

GUSTERSON, B.A., MITCHELL, D., WARBURTON, M. & SLOANE, J. (1982). Immunohistochemical localization of keratin in human lung tumours. *Virchows Arch.* (Pathol. Anat.) **394**, 269.

GUSTERSON, B.A. (1984). Precancerous changes in the lungs of the potential of cells to have modulated phenotypes. In: *Precancerous States.* (Ed. Carter), London: Oxford Univ. Press, ch. 6.

KREYBERG L., LIEBOW, A.A. & UEHLINGER, E.A. (1967). *The Histological Typing of Lung Tumours.* Geneva. World Health Organization.

MATTHEWS, M.J. (1979). Effects of therapy on the morphology and behaviour of small cell carcinoma of the lung—a clinico-pathological study. In: *Lung Cancer: Progress in Therapeutic Research.* (Ed. Muggia & Rozencweig), New York: Raven Press, p. 155.

McDOWELL, E.M., McLAUGHLIN, J.S., MERENYL, D.K., KIEFFER, R.F., HARRIS, C.C. & TRUMP, B.F. (1978). The respiratory epithelium V. histiogenesis of lung carcinomas in the human. *J. Natl. Cancer Inst.* **61**, 587.

McDOWELL, E.M., WILSON, T.S. & TRUMP, B.F. (1981). Atypical endocrine tumours of the lung. *Arch. Pathol. Lab. Med.*, **105**, 20.

MITCHELL, D.P. & GUSTERSON, B.A. (1982). Simultaneous demonstration of Keratin and mucin. *J. Histochem Cytochem.*, **30**, 707.

RABER, M., BARLOGIE, B., & FARQUHAR, D. (1980). Determination of ploidy abnormality and cell cycle distribution in human lung cancer using DNA flow cytometry. *Proc. Am. Ass. Cancer Res.*, **21**, 40.

SHORTHOUSE, A.J., CARTER, S.M. & ELLISON, M.L. (1982). Tumour marker production in human bronchial carcinoma xenografts. *Oncodev. Biol. Med.*, 3 273.

SHORTHOUSE, A.J., PECKHAM, M.J., SMYTH, J.F. & STEEL, G.G. (1980). The therapeutic response of bronchial carcinoma xenografts: a direct patient xenograft comparison. *Br. J. Cancer,* **41** (Suppl. IV) 142.

VINDELOV, L.L., HANSEN, H.H., GERSEL, A., HIRSCH, F.R. & NISSEN, N.I. (1982). Treatment of small cell carcinoma of the lung monitored by sequential flow cytometric DNA analysis. *Cancer Res.*, **42**, 2499.

WARENIUS, H.M. (1980). Identification and separation of mouse and human components of heterotransplanted human tumours. Ch. 19 In: *Immunodeficient Animals for Cancer Research.* (Ed. Sparrow) Macmillan Press Limited.